# Gelatin as a Photosensitive Material

**DOI:** 10.3390/molecules23082064

**Published:** 2018-08-17

**Authors:** Sergio Calixto, Nina Ganzherli, Sergey Gulyaev, Susana Figueroa-Gerstenmaier

**Affiliations:** 1 Research Department, Centro de Investigaciones en Optica, Loma del Bosque 115, Leon 37150, Mexico; 2 Department of Solid State Electronics, Ioffe Institute, 194020 St. Petersburg, Russia; nina.holo@mail.ioffe.ru; 3 Institute of Physics, Nanotechnology and Telecommunications, Peter the Great St. Petersburg Polytechnic University, 195251 St. Petersburg, Russia; Gulyaev@rphf.spbstu.ru; 4 Departamento de Ingenierias Quimica, Electrónica y Biomedica, Division de Ciencias e Ingenierias, Universidad de Guanajuato Campus Leon, Loma del Bosque 103, Leon 37150, Mexico; sfigueroa@ugto.mx

**Keywords:** gelatin, photosensitive materials, silver halide photographic emulsion, dichromated gelatin, selective tanning, short-wave UV radiation, photodestruction, diffraction efficiency, dyed gelatin, holographic structures, Weigert effect

## Abstract

Because this issue journal is dedicated to Gelatin, here we present a few applications of gelatin in the field of optics. Optics is the science that studies the production, propagation, interaction and detection of light. Various materials sensitive to light (photosensitive) are used for detection of light, such as photomultipliers, CCDs, crystals, two dimensional (2D) materials and more. Among the 2D materials, the most popular for several centuries has been gelatin based photographic emulsion, which records spatial distributions of light. More recently (1970), films made of Gelatin with Dichromate (DCG) and dyes have been used. We describe some characteristics and applications of these two photosensitive materials. We also describe examples where gelatin is used as a Relative Humidity (RH) sensor and in the fabrication of optical elements based on gelatin. This article is intended for researchers outside the optics community.

## 1. Introduction

Gelatin is a material that can be applied in many fields. For example, in food (confectionery, meats, bakery products, dairy products, beverages), health and nutrition (bone and joint health, beauty, calorie management), pharmaceuticals (hard and soft capsules, vaccines, tablets, absorbable hemostats), specialties (photographic gelatin, ballistics, lubricants, fuels, release agents, ignites), fats, proteins and minerals (pet food, fertilizers, live stocks) and more [1].

Regarding the physics-optics fields, gelatin can be applied in the fabrication of photosensitive materials. Undoubtedly the best known sensitive material is the photographic emulsion [2,3]. Light sensitive material in the emulsion is silver halide but it should be used with a binder that is gelatin. Some other binders, like polyvinyl alcohol, have been used but the better is gelatin. Other photosensitive material in the physics-optics fields is Dichromated Gelatin (DCG). Since its use in the printing industry for over a century ago it has shown applicability. Perhaps the last successful application is in the fabrication of holograms.

The photographic emulsion has been developed since the end of the 18th century. Many researchers (amateurs and professionals) and companies have made contributions to the development of photographic emulsions through the centuries. It is a theme that has been described in many books, articles and more. Here in this article we just mention a few applications of gelatin in photographic emulsion, DCG in holography and fabrication of micro-optical components. This article is devoted to researchers or people not acquainted with physics-optics. We give a brief overview of the potential that has gelatin in the photosensitive materials. For those interested in learning more details on specific themes can consult the references. Possibly they could gather more information by using searching and databases engines (Google, Scopus, Optical society of America OSA, SPIE, MDPI, Journals: Optics and Spectroscopy, J. of Optical Technology, etc.) and give the word “gelatin” to the searcher. Besides the work done in the “western world,” a great deal of interesting research has been conducted in the former USSR. It is good to search those references, for example [4,5,6]. More recently a Review of photosensitive materials for holography has been published [7]. Besides the gelatin based materials, the review includes photopolymers, photochromics, photorefractive, liquid crystals and more.

Section 2 shows the use of plain gelatin films and gelatin mixed with colorants. Section 3 mentions the applications of gelatin as a part of the photographic emulsion and applications of it in the fabrication of some optical elements. Section 4 describes the mixture of gelatin with dichromates (DCG), the use of undeveloped DCG films, dyed DCG, the Weigert effect in gelatin mixtures, DCG in the fabrication of solar concentrators and more. In each theme, we have mentioned some references that show part of the work developed by different groups. We do not show all the references to make this information manageable.

## 2. Plain Gelatin

### 2.1. Gelatin Chemical Characteristics, Fabrication Process, Environmental Stability (pH, Temperature, Humidity, Thixotropy, Ultrasound)

Gelatin or gelatine is an important functional biopolymer widely used in foods to improve elasticity, consistency and stability. It is also used in pharmaceutical drugs, vitamin capsules, photography and cosmetic industry. Gelatin is a protein made by the thermal denaturation of collagen. It is a colorless or slightly yellow substance. Commercially is available as a solid and transparent, brittle, odorless and tasteless granule, sheets, flakes or powder, soluble in hot water, glycerol and acetic acid and non-soluble in organic solvents. Gelatin swells and adsorbs 5–10 times its weight of water to form a gel. Gelatin forms a gel in water at a minimum concentration of 0.5% and at pH range from 4 to 8 [8]. Gelatin is widely used as a food ingredient and is also used as a gelling agent forming transparent elastic thermos-reversible gels on cooling below about 35 °C. Additionally, due to its amphiphilic nature, it has emulsification properties and foam-stabilizing properties.

The source of gelatin from animals are hide and bone and from vegetables are starch, alginate, pectin, agar and carrageenan but their gels lack the elastic properties of the gelatin that comes from animals. Gelatin is a mixture of peptides and proteins produced by partial hydrolysis of collagen extracted from the skins, bones, tendon and white connectivity tissues of animals such as domesticated cattle, chicken, pigs, fish and even some insects. During hydrolysis, the molecular bonds between individual collagen strands are broken down into smaller molecules. Photographic and pharmaceutical grades of gelatin come more often from cattle bones and pig skin [9,10].

Gelatin is a heterogeneous mixture of high molecular weight polypeptides and an important hydrocolloid. It differs from other hydrocolloid because most of them are polysaccharide, whereas gelatin is a digestible protein containing all the essential amino acids except tryptophan [11,12]. The amino acid composition, particularly with respect to proline and hydroxyproline, can vary between species as a result of exposure to a wide range of environmental conditions, in particular, temperature.

As we already mentioned, gelatin is prepared by the thermal denaturation and physical and chemical degradation of collagen. Collagen is the most abundant structural protein in both vertebrates and invertebrates and constitutes approximately 30% of an animal’s total protein. Gelatin in a dry form consists of 98–99% protein. The molecular weight of these large protein structures typically ranges from 20,000 to 250,000 g/mol, with some aggregates weighing in the millions. The chemically structure of gelatin is described by a linear sequence of amino acids. It is always written from the –NH_2_ end to the –COOH end by convention. The predominant amino acids are glycine, proline and hydroxyproline. As a result, gelatin contains relatively high levels of these amino acids: glycine 26–34%, proline 10–18% and hydroxyproline 7–15%. Other significant amino acids are: alanine 8–11%, arginine 8–9%, aspartic acid 6–7% and glutamic acid 10–12%. The water content will vary between 6–9%.

In gelatin manufacture, two methods are usually used: the acid and the alkaline processes (in the pretreatment part) to produce type A and type B gelatins, respectively. In the acid one, pigskin with an isoionic point of pH = 7 to 9 is used. In an alkaline method, asparagine and glutamine residues are converted to their respective acids and results in higher viscosity with isoionic point of 4.8 to 5.2 (pH). The functional properties of gelatin are related to their chemical characteristics. The gel strength, viscosity, setting behavior and melting point of gelatin depends on its molecular weight distribution and amino acid composition, the imino acids proline and hydroxyproline are important in the renaturation of gelatin subunits during gelling. As a result, gelatin with high levels of amino acids tends to have higher gel strength and melting point.

The criteria for good food-grade gelatins are not as demanding as those for photographic gelatin. Viscosity and gel strength are the main physical properties for grading any gelatin under carefully standardized conditions. Viscosity is determined at 60 °C at a concentration of 6.67% (*w*/*w*) air-dried gelatin. Gelatin forms gels similar to those of carbohydrates by forming a micro-structural network. It is unique in that, at concentrations as low as 1.0% it will form a thermoreversible gel. The gel converts to a solution as the temperature rises to 30 °C to 40 °C, thus gelatin gels tend to melt in the mouth. The melting point is the temperature at which a gelatin gel softens sufficiently and allowing carbon tetrachloride drops to sink through it. Factors such as the maturing temperature and the concentration of the gelatin gel tend to affect its melting point. The setting point of a gelatin solution is dependent on its thermal and mechanical history. Higher setting temperatures are encountered when the solution is cooled slowly in comparison to rapid chilling. Mechanical action hinders or delay setting.

Gelatin solutions have properties of thixotropy. This means that some jellies and gels, such as gelatin, agar-agar, hydrate of iron oxide and others, under mechanical action (shaking, stirring) are able to liquefy, that is go into sols, which in a state of rest again go into jelly. These transformations are reversible and can be repeated an unlimited number of times.

At the beginning of the 20th century (1930) it was shown that under the influence of ultrasound the viscosity of solutions of many macromolecules decreased. When ultrasound stops, the viscosity increases partially. The ultrasound method overcomes the Van der Waals energy interactions and leads to the rupture of macromolecules. Among materials that have been modified, using ultrasound, are gelatin, agar-agar, starch, rubber, gum Arabic and others.

Ultrasound is also used for solution emulsification. Emulsions can be made with vegetable or animal materials. Gelatin is the stabilizing and emulsifying agent. Under ultrasound process the gelatin structure is destroyed and this contributes to the stabilization of the emulsion because individual fat droplets percolate into the cells of the continuous grid. The maximum emulsifying efficiency is observed with gelatin contents in the range 0.25% to 1.0%. The use of high gelatin concentrations is impractical. Emulsions can be fabricated by means of mechanical dispersion methods but it is time consuming. However, when ultrasound is used the emulsions are fabricated quickly and the emulsions are more stable during long term storage.

The effect made by ultrasound on gelatin is, to some extent, equivalent to the damaging made by short-wave UV radiation. However, the ultrasound effect is a drawback for holographic plates because it should be carried out when gelatin is in the form of a solution. This is not acceptable in the holographic developing step because the ultrasound process will destroy the pattern recorded by the gelatin film when it was exposed to the interference lines formed by light. Besides, if the ultrasound process does not destroy the patterns, it will lead to the distortion of the regular structure recorded by the hologram film. However, the idea is original and, maybe in the future, some research could be developed and use the gelatin thixotropy in combination with the ultra sound method to improve gelatin films.

### 2.2. Gelatin as a Mid-Infrared Recording Medium

Gelatin is transparent to visible light. However, films with a thickness of about 90 µm only show about 10% transmittance for mid-infrared light (λ = 10.6 µm). For thicker films, mid-infrared light is highly absorbed. This characteristic has been used [13] to fabricate surface relief gratings. Thin films of gelatin having thicknesses from about 10 µm to about 50 µm were made and glued to O-rings. Then they were placed in a two-beam interference configuration which gives a sinusoidal spatial intensity pattern. A CO_2_ laser was used (λ = 10.6 µm). These films recorded the sinusoidal pattern. At the same time light from a He-Ne laser was sent to the recording area. It was found that through exposure time relief gratings were recorded. First order intensity was monitored through the exposure. Diffraction efficiency values of about 30% were attained. These recorded gratings were permanent. Their profile was studied with an interference microscope and resulted to be sinusoidal. Besides the interference gratings, holograms were also recorded. The sensitivity of the films resulted to be about some 1 J/cm^2^.

Besides the test of the diffraction gratings, with light from a He-Ne laser, they were tested by sending IR light from a CO_2_ laser. To increase gratings IR reflectance their surface was coated with an aluminum film. The result was that diffracted orders appeared.

### 2.3. Gelatine as Relative Humidity Sensor

**Figure 1:**
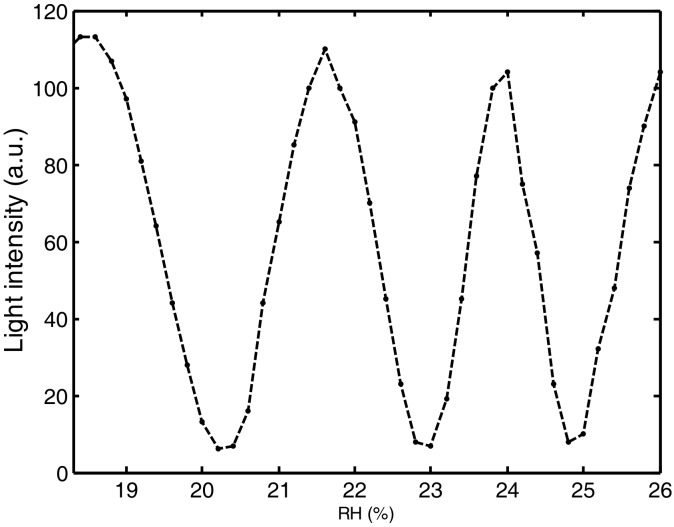
Light intensity as a function of Relative Humidity when a thin layer of gelatin was used as the humidity sensor.

We have seen in Section 2.1 that gelatin is a complex chain of amino and imino acids linked together in a partially ordered fashion by polypeptide bones. When water vapor is absorbed by (desorbed from) the gelatin films its thickness changes. Film thickness will be minimum when the film is dry and maximum when is swollen. When gelatin is dry its refractive index is about 1.5. But when water vapor is absorbed by the gelatin its refractive index will diminish because the water refractive index is 1.33. These thickness and refractive index changes can be detected with a Mach-Zehnder interferometer. In reference [14] a method to measure Relative Humidity (RH) using a gelatin thin film as sensor element is mentioned. Authors used thin gelatin films of about 24 µm glued to an O-ring. To test the films a climatic chamber was used. In this chamber, a Mach-Zehnder interferometer was placed. A He-Ne laser was used as the light source. This interferometer comprises two beams of light. One is called the reference and the other the testing beam. At the output of the interferometer both beams are superposed forming a sinusoidal interference pattern. The gelatin film is inserted in the testing beam. The RH in the chamber can be modified. Gelatin film will respond to the changes in RH by changing its refractive index and thickness, as we have said. These changes will make that the interference pattern present a lateral displacement. By using a light point sensor, it is possible to monitor the displacement, Figure 1. Thus, we have a plot relating the intensity of the interference pattern as a function of RH. This is the calibration plot. Gelatin films could present different physical characteristics that will affect the RH sensitivity of the films.

Besides the use of gelatin as RH sensor in a Mach-Zehnder interferometer, gelatin has been used as the sensing element when optical fibers are used. In reference [15] is described a RH sensor based on “single mode-multimode-single mode” fiber structure (SMS). The multimode fiber is polished to remove the cladding and part of the core, remaining a fiber structure with a “D” shaped multimode profile. Then very thin gelatin layers are coated on the multimode fiber section by means of the Dip Coating technique. They tested coatings with 3 and 6 layers. In the experiment one end of the SMS was connected to a supercontinuum light source and the other to an Optical Spectrum Analyzer (OSA). The SMS structure with the gelatin layers was placed in a climatic chamber where it was possible to control temperature and RH. If RH raises gelatin refractive index decreases and the losses in the light transmission will increase. By monitoring the losses for each wavelength from about 1490 nm to about 1510 nm they got plots of Transmission losses as a function of wavelength and the parameter will be the RH. These are the calibration plots. A response time of 1 s was mentioned. Besides the study just mentioned, reference [15] describe other application of gelatin films together with optical fibers. For more information about gelatin applications with optical fibers see the reference section of reference [15].

### 2.4. Gelatin with Colorants (Dyed Gelatin)

We have seen that gelatin films transmit well light with wavelengths between 400 nm and about 800 nm. Thus, to make gelatin films sensitive to visible light a dye should be added. This process was exposed in Reference [16] by the group of Prof. Sirohi, in which authors mention the use of an organic eosin dye embedded in thin layers (8 µm) of gelatin. Eosin dye belongs to the family of xanthene dyes. Authors recorded sinusoidal interference patterns (λ = 532 nm, Nd:YAG laser) with a period of 5.7 µm. The result was the formation of surface relief gratings. When studied with an Atomic Force Microscope these gratings showed a sinusoidal relief. The depth of the relief was about 70 nm for gratings having 3% diffraction efficiency (DE).

Another study that involved the use of dye incorporated in gelatin is described in References [17] and [18] by the group of Prof. Pantelic. There, authors describe a mixture of gelatin, tot’hema and eosin to render gelatin sensitive. Tot’hema is a drinkable solution used in medicine for curing anemia. Eosin is an organic dye used in medicine too. It shows a maximum absorption in the green part of the spectrum. Gelatin layers were fabricated by the gravity settling method. 100 µm thick layers were made. A Nd:YAG laser was used to make negative microlenses. Authors showed parabolic profiles. The process to form the lens is thermal. Radiation from the laser is completely absorbed by the film. As energy is absorbed an increase in the layer temperature is present until it reaches the gelatin melting point. At this stage, the gelatin becomes liquid. By thermocapillary forces the liquid flows and the formation of a dip occurs.

The recording of holograms considers the interference of two light beams, one that comes from the object (object beam) and another one called reference. Here it is supposed that both beams have the same linear polarizations which is perpendicular to the plane of incidence. However, object beam could present any polarization state, that is linear, circular or elliptical. Thus, a recording medium capable of recording the polarizing pattern should be used. Once the recording is done the hologram is illuminated with the reference beam and the object beam is generated. It will present the same polarization state that light coming from the object had. This is called polarization holography. The group of Prof. Ebralidze (Georgia) [19,20,21,22,23] developed a technique where authors used dyed gelatin to record polarizing holograms. Azo-dye-colored gelatin films (methyl-orange, methyl-red and other dyes) were prepared on glass plates. Then they were illuminated with a mercury lamp. This light induced anisotropy. Light is absorbed by the dichroic molecules whose axes are oriented along the polarization vector. These molecules become centers of photoinduced crystallization. The grain concentration is proportional to the light intensity. Authors developed theory and experiments. In references [19,20,21,22,23] are mentioned some articles of the group but authors published more information.

## 3. Gelatin in the Photographic Plate (Mainly Holography)

### 3.1. Photographic Plate in Holography

In 1674 Christoph Baldwin produced calcium nitrate by highly heating a mixture of chalk and nitric acid [24]. After drying the mixture, he found that it was luminous. He called the mixture “Phosphorus” (carrier of light). Later in 1721 Heinrich Schulze attempted to reproduce Baldwin’s experiment. At one time, he made a mixture containing nitrate of silver, chalk and nitric acid. He though this will dissolve chalk. When, after heating, he exposed accidentally this silver mixture to light he noticed that silver salts were sensitive to light. He discovered the sensitiveness to light of the silver salts. Later the based silver salts photosensitive wet plates were proposed to record images (Daguerrotypes). In 1850 Poitevins did the first experiments that used gelatin as a binding element for silver salts to make sensitive emulsions but were unsuccessful. Until 1871 Maddox mentioned the use of gelatin as a binder for the silver bromide emulsions. This was the first successful dry emulsion made with gelatin silver bromide.

In the photographic plate three silver halides can be used: silver chloride, silver bromide and silver iodide. Some of them give more sensitivity to the plate. Grain sizes vary from about 10 nanometers to a few micrometers. The sensitivity of the plate can go from the ultraviolet light to the green part of the spectrum depending on the mixture of silver halides. By adding dyes, it is possible to cover the visible spectrum and infrared light. For a more in-depth description of the photographic plate fabrication, sensitivity, characterization, emulsion structure and more, the reader could consult the references [2,25].

Through some centuries the photographic emulsion was used to record mainly photographs and radiographies. Much scientific work was developed to offer very good photographic plates. However, when holography was developed by Dennis Gabor, in 1948, new mixtures of the photographic emulsion were fabricated. Holograms are made by recording interference light patterns. These patterns are produced when light from an object interferes with other beam called reference beam. These beams are coherent. They usually interfere at an angle. The number of interference lines could be between about a few lines per millimeter (L/mm) to about 5000 L/mm. Thus, new studies were made to find silver halide emulsions that fulfilled the requirements of holography. Among those requirements the recording medium should present high sensitivity, high spatial resolution, linearity in response, low noise, this implies that that the material should not present grain structure, if there are grains they should be small, availability in different formats, low cost and long-term stability. Several references mentioned the available commercial emulsions at that time (1970) and later [25,26,27,28,29,30]. Unfortunately, some of those emulsions are not produced anymore.

Currently (2018) one supplier of photographic papers and holographic plates is Slavich [31]. It has the following emulsions: PFG-01 (red sensitive plates and films), PFG-03M (red sensitive plates), PFG-03C (panchromatic plates and rolls), DCG plates (Blue green sensitive, PFG-04), VRP-M and VRP silver halide plates (green sensitive).

At present (2018) a few groups make studies of the actual silver halide plates. One is the group of Prof. Belendez in Alicante, Spain. Authors have made different studies of the PFG-01 and the BB-640 plates [32,33,34]. More references to their work can be found. Other group is in Saint Petersburg National Research University of Information Technologies, Mechanics and Optics (ITMO), St. Petersburg, Russia [35]. Authors have studied new visible light optical sensitizers for PFG-03 plates.

Regarding the use of silver-halide plates when near-infrared and mid-infrared light is used, a study [36] shows that by the use of triethanolamine and a thermal cycle it is possible to extend the sensitivity range of silver halide plates to record light with a wavelength of 1.7 µm. Other study [37] shows the use of silver halide films using light with a wavelength of 10.6 µm. See the references therein to find more information about the use of silver halide films with infrared radiation.

### 3.2. Short Wavelength Ultraviolet Method (SWUV) Used to Fabricate Holographic Structures

#### 3.2.1. Introduction

Gelatin is the main component of the two most common photosensitive media used in optics (comprising holography): silver halide photographic emulsions and layers of Dichromated Gelatin (DCG). Therefore, the efforts of researchers in the holography field have been aimed at the development of methods that change the physical-chemical properties of gelatin in accordance with the recorded interference pattern. Gelatin processing methods involve two main types of effects: selective structuring and destruction of gelatin molecules. When they are applied, separately or together, they give rise to a variety of methods in the fabrication of high performance phase holographic structures on gelatin-containing photosensitive media.

The formation of the phase holographic structure is associated with the “development”—an operation that allows the modulation of the physical and chemical properties of gelatin. There are two kinds of the development: equilibrium and non-equilibrium. The non-equilibrium “development” involves the selective structuring (tanning) of the gelatin layer. When silver halide plates are used they suffer the tanning changes through the bleaching chemical process. However, if DCG is used the process is done photolytically. In both cases the non-equilibrium “development” is done by the rapid dehydration with isopropanol of the gelatin layer. This includes the method of creating “microcavity holograms” [38,39] and the Silver Halide Sensitizing Gelatin method (SHSG) [40,41]. The non-equilibrium development methods are extremely sensitive to the processing conditions and the type of silver halide photoemulsion or DCG used. The application of these processes allows the fabrication of purely phase volume holograms.

#### 3.2.2. The SWUV Method among Other Methods of Obtaining Phase Holographic Structures

The equilibrium “development” is associated with the slow drying of a gelatin layer in the air. This means that the use of isopropanol is not required. Equilibrium development methods are associated with either selective tanning, when silver halide emulsions are bleached [42], or selective gelatin photodestruction of the gelatin-containing sensitive media [43,44]. When these processing methods are applied good results are obtained regardless of the type of the gelatin containing sensitive medium and processing parameters. The application of these methods allows the creation of purely phase relief holographic structures.

**Figure 2:**
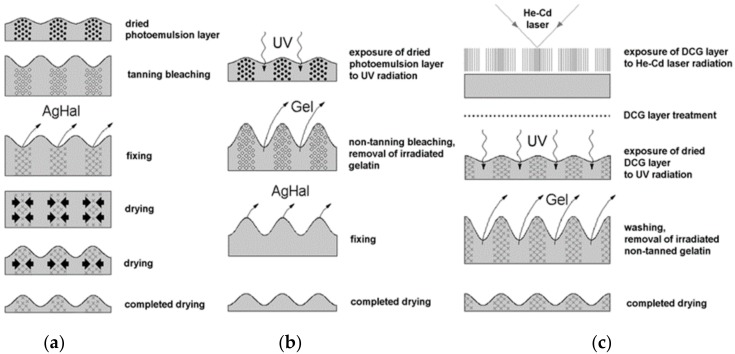
Creation of surface relief (**a**) during selective tanning and by destructive effect of UV radiation (**b**) on silver-halide photoemulsions and (**c**) on layers of DCG.

The formation of the surface relief during selective tanning of silver halide photoemulsion is due to the tension forces arising in the process of the photoemulsion drying. The redistribution of gelatin leads to the formation of crests of surface relief at tanning areas, that is at the areas with the greatest density of a Silver Image (SI) (Figure 2a) [42].

As an organic substance, gelatin absorbs UV radiation well. This is the basis for the method of the destructive action of short-wave UV radiation, with a wavelength less than 250–270 nm, on gelatin (SWUV method) of the silver halide photoemulsion [43]. In this case, the primary SI serves as an effective screen that modulates the intensity of the UV radiation. The destructive effect of UV radiation leads to breaking main bonds in long chains of gelatin macromolecules and their fragmentation and solubility in aqueous solutions (see Figure 2b).

In the case of DCG, the physical-chemical properties of gelatin are changed directly by the holographic recording of the interference pattern due to selective laser light tanning in the presence of dichromates (Figure 2c) [44]. Thus, a structuring takes place, that is, the creation of a large number of cross-links in the maxima of an interference pattern. A large number of cross-links prevents the fragmentation of gelatin macromolecules under the effect of UV radiation and their dissolution in water. Thus, the crests of the surface relief after the water procedure are formed in the maxima of the interference pattern. Both main effects on gelatin: selective structuring and destruction, are consistently applied for the formation of a phase hologram on DCG.

All the methods of creating relief-phase holographic structures by means of the equilibrium type of the development allow one to obtain a significant depth of the surface relief in the range of spatial frequencies up to hundreds of lines/mm (L/mm).

#### 3.2.3. Regular Holographic Structures Obtained by the SWUV Method

The DE of relief-phase structures obtained by holographic methods is largely determined by the height (depth) of the surface relief *h* measured as the total difference between the crests and valleys. Since the range of recorded spatial frequencies does not exceed several hundred L/mm, the diffraction of light can be described by the Raman-Nath approximation [45,46,47]. According to this approximation, the DE $\eta_{1}$ of a transmission structure with a sinusoidal relief profile, in the first diffraction order, can be represented as:

(1) $$\eta_{1}\;=J_{1}^{2}\left[\frac{\pi {\;(\;n}_{0}\;-\;1\;)\;h}{\lambda }\right]\times 100\%\;$$

where the amplitude of the phase modulation of the illuminating beam is in the argument of the Bessel function *J*_1_. The square of the Bessel function becomes the maximum value of ~ 0.34 when the value of its argument is approximately equal to π/2:

$$\left[\frac{\pi {\;(\;n}_{0}\;-\;1\;)\;h}{\lambda }\right]\approx \frac{\pi }{2}\;$$

Since gelatin has a refractive index *n*_0_ close to 1.5, then the height of the surface relief *h*, providing a maximum intensity value of the first diffraction order, is approximately equal to λ. This value can be considered as the criterion for the surface relief depth that is necessary for the effective diffraction of the regular periodic structure.

Table 1 presents the main parameters of the regular holographic structures on silver halide photoemulsions of different types and DCG layers, created in the laboratory, by the SWUV method [43,44,48,49,50,51,52].

As can be seen from the Table 1, a large value of the depth of the surface relief provides a high DE of regular structures, approaching the theoretical limit for thin phase holograms. This indicates the possibility of creating high-performance regular holographic transmission structures for the visible and near-infrared light spectrum.

#### 3.2.4. Random Phase Structure Obtained by the SWUV Method

The determination of the required depth of the surface relief for random holographic structures (diffusers) is somewhat different than the one presented in the preceding section. For practical applications of random structures, it is necessary to ensure a minimum proportion of the non-scattered light component (zero diffraction order) that passes through the diffuser. According to the theory of light scattering on large-scale inhomogeneities in the Kirchhoff approximation, if the distribution of the relief height is described by the Gaussian function [53], the amplitude reflection coefficient of the non-scattered component is equal to:

(2) $$V(\psi )=\exp(-2k^{2}\sigma^{2}\sin^{2}\psi )\;$$

where k = 2π/λ is the wave number, λ is the wavelength of light, σ is standard deviation of the relief height and ψ is the slope angle of the beam. For transmission structures [52] at ψ = 90°, Formula (2) is converted to:

(3) $$\eta_{0}=\exp\left[-\frac{{4\pi }^{2}}{\lambda^{2}}{{(n}_{0}-1)}^{2}\sigma^{2}\right]\times 100\%\;$$

where $\eta_{0}$ is the relative intensity of a zero-order beam as a percentage of the incident beam, n_0_ is the mean refractive index of gelatin equal to 1.53 (for λ = 0.6328 µm). The small values of the relative intensity of the non-scattered component, for example, $\eta_{0}$ ≤ 0.1%, are achieved with values of the standard deviation of the surface relief height σ ≥ 0.5 µm. The last inequality can serve as the criterion for the efficiency of the transmission scattering structure.

Table 2 presents the main parameters of holographic diffusers obtained on PFG-01 plates when using the SWUV method [54]. The SWUV method is able to provide the value of the standard deviation of the surface relief height (sample 1), which is necessary to achieve the low value of the intensity of scattered light components $\eta_{0}$. The low values $\eta_{0}$ (samples 2, 3) can be also obtained at the values of standard deviation σ that are significantly lower than the values required by the theory. This can be explained by the fact that some samples may maintain certain regularity in the structure depending on the way the diffusers are produced.

Thus, the SWUV method proved to be applicable for the creation of efficient random and pseudorandom structures such as narrow directed diffusers operating in the visible spectrum range [54,55,56,57].

#### 3.2.5. Creation of a Large Depth Surface Relief and the Phenomenon of the Structure Period Doubling

The effective absorption of the short-wave UV radiation by gelatin is limited to a narrow near-surface layer due to the strong self-absorption of gelatin [58]. Thus, the photodecomposition of gelatin and its transformation into a soluble state, is a purely surface process covering only the depth of 1–2 µm of the total thickness of commercially produced photolayers that is equal to 5–18 µm. Therefore, the required original thickness of the silver halide photographic material can be significantly reduced. For example, high values of DE $\eta_{1}$ can be obtained when SRBSh photoemulsions are used (Table 1, #9). This emulsion has a thickness of *T* = 1.8 µm.

**Figure 3:**
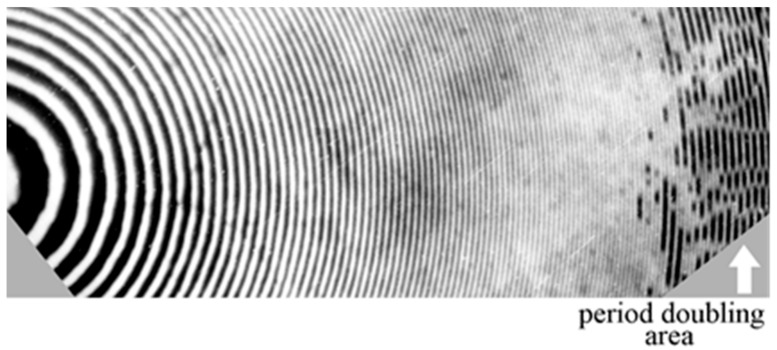
Microphotograph of holographic Fresnel Zone Plate, including the area with period doubling.

There are some applications where the holographic structures should present significantly larger surface reliefs than the ones given in Table 1. These applications comprise the use of structures working with infrared light, structures that should present higher diffractive orders, or structures that can be used as a mold to transfer their holographic characteristics to other substrates. The repetition of the treatment cycle “UV radiation–washing–air drying,” for the VRL photoemulsion, allowed us to increase the height of the surface relief h at low spatial frequencies (ν ≤ 30 L/mm) up to the values of 5–8 µm. In the places with the lowest SI density it is possible to obtain “windows” where the layer thickness tends to zero [48]. That is, there is no gelatin in those places. However, in the area of higher spatial frequencies (ν > 40–60 L/mm), a very interesting physical phenomenon was discovered: the doubling of the period of the holographic structure (the formation of spatial subharmonics), Figure 3 [48]. The significant increase of the total UV exposure time and the number of repeated dryings generate two processes: destruction in the places with the lowest density of SI and photostimulated tanning (structuring) in the same places. As a result, the holographic structure loses the stability. The tension forces initiate the pairwise union of neighboring relief crests and the violation of the spatial symmetry of the structure. A similar example of the spatial self-organization and formation of domain structure caused by the instability of the thermodynamic system is present in the field of dynamic holography when photorefractive crystals are used [59].

The phenomenon of period doubling, when ultra-deep reliefs are obtained, can be avoided if a multi-cyclic treatment is replaced with continuous aqueous etching of gelatin while irradiating samples with a mercury lamp [48].

#### 3.2.6. SWUV Method and Ultra-Thin DCG Layers

The concept of the surface action of short-wave UV radiation on gelatin is quite applicable to the layers of DCG. Instead of the modulation of the intensity of UV radiation by a SI, we have here a variable layer tanning (Figure 1c), that modulates the destructive effect of short-wave UV radiation on gelatin in the upper layers. A significant decrease in the thickness of the DCG layer (Table 1, #11, 12) [51,52] up to the values of the order of λ provides high values of the DE $\eta_{1}$ that are close to the theoretical limit for thin holograms. In the case of ultra-thin DCG layer (*T* < 1 µm), for a sufficiently long UV irradiation time, gelatin-free “windows” appear. Multiple repetition of the cycle “UV radiation–washing–air drying” for the layers with the thickness *T* < 3–6 µm leads to the same result. This fact allowed one to realize the effective transfer of holographic relief structures from the DCG layer onto a PMMA substrate [52]. In this case the sequential application of the DCG treatment and the resists processing are used (Table 1, #12).

### 3.3. Other Applications of the Photographic Plate

Nowadays (2018) the photographic plate has been replaced as light sensitive material by CCDs and other electronic devices. However, photographic emulsions are applied in fields like recording of landscapes and portraits, motion pictures, aerial photography, microfilm, traffic and surveillance, nuclear physics and more. For more information on the actual commercial emulsions please consult the web pages of Kodak, Agfa-Gevaert, Fujifilm, Ilford and Slavich companies to mention but a few.

The photographic plate can be used to make relief micro-optical elements. By pure relief is meant that there are variations in the height of the gelatin. Altman [60] mentioned that when common developers were used the height of the relief image on the plate was of the order of 1 µm when the silver density was 4.0. But still higher relief images were generated when tanning bleach was used. Relief images higher than 3 µm were obtained. Other studies mention the use of PE-2 photographic plates [61]. Based on this method it is possible to fabricate relief surface zone plates [62] and microlenses [63].

Cloud chambers and bubble chambers have been used for visualizing the passage of ionizing radiation. The discoveries of the positron (1932) and the muon (1936) were done with cloud chambers. These chambers use photographic emulsions. More recently an experiment was done to detect Tau neutrinos originated from Muon neutrinos [64,65,66,67]. Protons were generated at the CERN facilities (Switzerland) and sent to carbon targets which produced pions and kaons. They decay to produce muons and neutrinos. To find the decay of Muon neutrinos into Tao neutrinos a special chamber was built in Italy, 730 km away. This consisted of an Emulsion Cloud Chamber (ECC) 10 m × 10 m × 20 m. The name of the project was Oscillation Project with Emulsion-tRacking Apparatus (OPERA). The basic ECC unit of OPERA detector is a brick that consists of a sequence of 57 emulsion photographic films interspaced with 56, 1 mm thick lead plates, packaged in a light tight container. The brick weights 8 Kg and have a thickness of 7.5 cm. The total number of bricks is 154,000 giving a total number of 10 million nuclear photographic films. These special films are made by Fuji Film co. Each film is made of two 44 µm thickness sensitive layer separated by a 205 µm thickness plastic base. The size of the grains in the photographic nuclear emulsion is between 0.1 µm and 0.5 µm. A detailed explanation of the nuclear photographic emulsion is given in reference [64]. See also the references therein.

## 4. Dichromated Gelatin (DCG)

### 4.1. DCG Characteristics and Sensitivity (UV and Visible Light)

A dichromated colloid consists of an organic colloid dissolved in a solvent that is usually water. To this solution a chromate or dichromate is added. They form photosensitive layers. Among the sensitizers are, for example, ammonium dichromate, potassium dichromate and sodium dichromate. All chromium compounds are poisonous. They can be absorbed through the skin and act as a protein precipitant. Photosensitive organic colloids can be divided in two general groups, proteins and carbohydrates. Gelatin, animal glues, albumin, casein and others belong to the first group. Gum Arabic and starch belong to the second group. The compound of gelatin and dichromates (DCG) is the most used in optics. When the colloid is exposed to light the colloids are hardened. In the dichromated colloids the photosensitive component, the dichromate, absorbs efficiently ultraviolet, violet and blue light. Maximum absorption is at a wavelength of 367 nm. Factors that affect the sensitivity are the dichromate concentration, value of the pH, layer thickness and moisture content, for example. A detailed description of dichromated colloids can be found in reference [68].

### 4.2. Undeveloped DCG

#### 4.2.1. Real Time Use of DCG

Holograms and diffractive elements made with DCG present very good diffraction efficiency. However, after the recording step DCG plates should be developed and this is a drawback because it takes time and plates should be displaced out of the recording configuration. There are holographic materials that can be used in real time but they are expensive, like the photosensitive crystals. However, it has been shown that DCG can be used in real time. DCG responds efficiently to ultraviolet (UV) and blue-green light. But it is almost insensitive to red light. Thus, the recording step can be made with UV-blue-green light and the reconstruction in real time can be done with red light. For example, using light from a He-Ne laser. The characterization of DCG working in real time was exposed in reference [69]. Diffraction efficiencies of about 0.2% were found. Gratings spatial frequencies ranged between a few hundreds to above 1000 L/mm. Applications in the fields of interferometry, object edge enhancement, character recognition, image subtraction and phase conjugation were presented.

Further studies of DCG plates used in real time were shown in reference [70]. Authors developed theory that predicted the behavior of diffraction efficiency as a function of time of exposure. The recorded gratings resulted to be pure absorption gratings. This phenomenon was present whenever the exposed plate remained in an environment with the same relative humidity with which the gelatin was sensitized, dried and exposed. However, when recorded absorption gratings were stored in an atmosphere with higher humidity a phase grating with more diffraction efficiency was present.

#### 4.2.2. Thick-Layered Self-Developing Dichromated Gelatin for Volume Hologram Recording

Two types of self-developing photosensitive materials, based on DCG, for real time volume hologram recording were created. The developing process of a hologram recorded in DCG requires some quantity of water. In one case, we produced a sandwich structure in which dichromate gelatin is found between two glass plates [71]. We call this layer “gel-like”. The process of layer manufacture was similar to the well-known technology of DCG [72]. The major difference is that the hologram is recorded directly in the moisture-saturated layer. The layer itself is a rather dense gel with a thickness ranging from 1 mm to 5 mm. The hologram can be recorded as easily as with a solid DCG film.

In another case, the self-developing glycerol-containing dry layers of DCG, with a thickness of 70–400 μm, were produced and studied [73]. Similar layers with a small thickness of 1–5 μm were described earlier in [74]. Glycerol serves as a plasticizer and is used to hold a certain amount of water molecules. These molecules are involved in the development of latent image due to the presence of hydrogen bonds. A hot solution of DCG with the addition of glycerol was poured on a glass substrate. The layers were gelled in a refrigerator for a day and then dried for several days at the room temperature. As a result, their thickness decreased by a factor of 5–6.

The main holographic parameters of the layers, “gel-like” and “glycerol containing”, were determined by recording holographic gratings using the symmetric scheme. The He-Cd laser (λ = 442 nm), with a power of 16 mW, was used as a light source. Holographic parameters of the gratings in DCG layers containing glycerol are better than those of the gel-like DCG. The maximum attained DE was 15–40% depending on the layer thickness and ammonium dichromate concentration. The sensitivity of a thick-layer glycerol-containing DCG is equal to about 6–10 J/cm^2^, which is close to the sensitivity of thick-layered gel-like gelatin (10 J/cm^2^). The thick-layered DCG containing glycerol is characterized by a virtually endless storage time for recorded holograms unlike the gel-like DCG that had the smallest lifetime of the recorded information (several hours). The main applications of the above-mentioned DCG volume media operating in real time are described in [75].

More studies of the mixture DCG, glycerol and methylene blue were done [76,77]. They characterized the films finding the optimal relation of glycerol and gelatin. The addition of glycerol decreased the achievement of the maximum diffraction efficiency.

### 4.3. DCG Used to Make Relief Lenses and Gratings

DCG is usually used to make phase elements, that is, modulation of the bulk is the base. However, it can be used to make relief optical elements like lenses and diffraction gratings. Reference [78] mentions the method for making microlenses and micromirrors. Unhardened layers with a thickness of about 8 µm were used. The optical recording configuration comprised the use of a white light source, a mask and a lens. The mask consisted of holes with different sizes. An image of the mask was formed over the DCG layer. Diameter of the holes images ranged between several hundred microns to some millimeters. Thus, this is a lithography method. After the recording, layers were developed with water and alcohol. At the end of the process relief microlenses and micromirrors were present. They were investigated with an interference microscope. The sagita value was a few microns. Lenses presented focal distances of some millimeters. Different types of masks were studied. Some had holes, as we have mentioned, others rings (annular design) and also masks with parabolic transmittance. When this last mask was used, the lenses gave the best image. Also array of microlenses were made by interference of three beams. These beams were not in the plane of incidence.

To commercialize holograms, it is common to copy the relief of master holograms onto different polymers. This copy process is called embossing or casting. A study was done to find if the master holograms could be made with DCG [79]. To characterize the DCG film, interference gratings with different spatial frequencies were recorded. DCG plates had a thickness ranging between 0.7 µm and 0.8 µm. Films were made by incorporating ammonium dichromate in a 2% gelatin solution. A He-Cd laser was used. After the exposed gratings were recorded the DCG plates were washed in water, the unhardened regions were dissolved and a relief grating appeared. It was found that DCG layers, show very low scattering but its spatial resolution fell when the spacing between fringes was in the order of a few microns.

The relief enhancement of recorded holograms can be made by means of biochemical etching. In reference [80] they used a proteolytic enzyme called Papain dissolved in water. Diffraction gratings on DCG were fabricated by contact-copying Ronchi gratings. These gratings consist of transparent and opaque strips with low spatial frequencies (0.25 L/mm). After the recording and developing steps they immersed the plate in a solution containing papain from 0.1 to a few units of %. They found that the relief depth was increased. Diffraction efficiencies rose. However, when gratings were made by recording a sinusoidal interference pattern, using an argon laser, the results were not so positive, spatial frequencies varied from 500 L/mm to 1500 L/mm. Besides this research with papain it seems in reference [81] they also used biochemical etching but the biomaterial is not mentioned.

### 4.4. Dyed DCG

As we have seen above the spectral sensitivity of DCG is limited to light with wavelengths less than about 540 nm. In the early 70s efforts were made to sensitize DCG films to red light [82,83]. One of those references mentions the use of thiazine and triphenyl methane family dyes. The former dye precipitated in the presence of ammonium dichromate solutions. The last family of dyes showed better compatibility with dichromate solutions. Used dye was Acid Fast Violet BG (AFV). They tested the DCG Dye sensitized plates to record gratings with 815 lines/mm and 1000 lines/mm and found a maximum diffraction efficiency of about 30% for layers having 6 µm thickness and about 50% for layers 20 µm thickness. A better method to sensitize DCG layers was proposed by Prof. Kubota [84,85,86,87,88,89,90,91] in 1976. Author used methylene blue (MB) as sensitizer. In the first reference, he describes the practical method for preparing DCG-dye layers. He found that the absorption spectrum of MB sensitized plates showed an absorption band at 372 nm, due to the ammonium dichromate and other two absorption bands were present at 620 nm and 670 nm that were due to the MB. 90% of light with 633 nm wavelength was absorbed in a 20 µm thickness layer. After testing the plates, he found a diffraction efficiency of about 88% for gratings that had 1050 L/mm. The energy required to achieve these efficiencies was 150–400 mJ/cm^2^. This energy is about 10 times the needed for conventional or DCG plates with no dye.

More studies and applications of MB dyed DCG were made besides the studies made by Prof. Kubota. For example, Changkakoti [92,93,94] performed systematic studies to investigate the influence of various relevant chemical and physical parameters on the diffraction efficiency of MB sensitized DCG plates. He studied: (a) the pH dependence of the developer, (b) the role of the external electron donor and (c) their storage life and processes of MB sensitized DCG plates. After him a study presented a new electron donor [95]. It was found that tetramethylguanidine (TMG) produced bright holograms when exposures in the order of 50 mJ/cm^2^ were given. Another study [96] mentions the role of the ratios and concentrations of prehardener, fixer and sensitizing solution as well as pH dependence and storage time.

Besides MB with dichromates other dyes of the xanthene group have been tested to record interference gratings. A study [97] mentions the use of Rhodamine 6G and Erythrosin B. A He-Ne laser giving light with a wavelength of 543 nm was used for the recording. It was found that sensitivity with these dyes was better than just DCG at the mentioned wavelength.

### 4.5. Weigert Effect in Gelatin Films

In this section, we will describe the formation of anisotropic plates that show the Weigert effect. Plates based on gelatin can be photographic emulsions, DCG-dye plates or just dyed gelatin plates.

Suppose that we have a silver halide emulsion and it is illuminated by natural white light until saturation occurs. Then it is developed to transform the silver halide into metallic silver. After this the plate is bleached and the opaque metallic silver is converted into a transparent silver-chloride emulsion. Then the plate is illuminated with a strong linearly polarized light. During the exposure, the illuminated areas will darken due to the instability of the bleached silver halide. If after the exposure we place the plate between crossed polarizers the illuminated areas will behave as a uniaxial (dichroic) medium. This phenomenon was described by F. Weigert in 1919 and it is known as the “Weigert effect”.

In a series of papers [98,99,100,101,102,103,104] Jonathan and May have exposed the results of experiments when they used silver-chloride plates. For example, they recorded two images in a silver-chloride plate. Then they observed the plate between crossed polarizers. This allowed a separate restitution of any of the two images. Another example is given when a transparency is placed in contact with the silver-chloride and illuminated with polarized light. Then the plate is placed between crossed polarizers and the image of the transparency is shown with its polarity. However, if the analyzer is rotated the birefringent parts will turn blue and the non-exposed ones will be white giving an appearance of a contrast reversal. Also with this method they found the difference between two transparencies.

The photoanisotropy phenomenon or Weigert effect can also be obtained with dyed dichromated gelatin layers. This application was described by Prof. Kakichashvili in a series of papers [105,106,107,108,109]. He used dyed-DCG plates. In the beginning, he used blocks that had embedded a photochromic substance—Trimethylspiranebenzopyran. He illuminated them with He-Ne polarized light and then he bleached them. He observed a photoanisotropy effect. He used this phenomenon as the basis of what he called Polarization Holography. That is, the recording of intensity, phase, wavelength and polarization of an object wave when interferes with a reference wave. In reconstruction, the object field together with its polarization is obtained. Later he proposed another photosensitive medium that was DCG with dyes. He mentioned the use of dyes from the triphenylmethane group. Some of them showed the capacity to produce the photoanisotropy or Weigert effect. Anisotropy was detected when samples were introduced in a polariscope. More recent applications of the Weigert effect are mentioned by Wardosanidze in Reference [110]. Another group that intensively worked in polarizing holography was led by Prof. Todorov [111,112,113,114]. They began using KCL:Na material containing Fa centers. Later they used mixtures of dyes and polymers. Another material that they developed, also based on gelatin, was mentioned in a patent [115]. There they mention the use of Arsenous sulphide (As_2_S_3_) dispersed in gelatin. Later they exposed a characterization [116] of the mixture where they show that dyed gelatin plates show an absorption edge shift when they were illuminated with linearly polarized light. More information can be found in reference [117].

Among the dyes mentioned by Kakichashvili was malachite green. Later more studies using this dye were presented in references [118,119,120,121,122,123].

### 4.6. DCG in Holographic Solar Concentrators

Holograms can be made to display images or to behave as optical elements. Among the optical elements are the lenses. In cases where a hologram behaves like a lens it is called a hololens. It can focus light or form an image. However, because holograms are based on the diffraction phenomenon if light with different wavelengths reach the hololens each wavelength will be focused in a different position.

If an object is placed outside the optical axis of an ordinary lens its image will be also outside the optical axis. This characteristic can also be presented by the hololens. It can be fabricated so as to work as an off-axis element. Thus, light that comes from a distant object will be focused outside the optical axis.

Light that comes from the sun can be considered as direct o diffuse. Sunlight presents many colors or wavelengths. Its spectrum is centered in the yellow part. The range of wavelengths in the solar spectrum mainly goes from about 0.3 µm to more than about 1.3 µm. This light can be converted to electricity by Photo Voltaic cells (PV). Each kind of these cells responds better to a certain range of wavelengths. Among the cells there are those made of Indium Gallium Phosphate (InGaP) and those made of Silicon (Si). The formers respond to wavelengths from about 0.3 µm to about 0.7 µm with peak absorption at about 0.65 µm. The last ones show a range from about 0.3 µm to about 1.1 µm with peak absorption at about 1 µm.

Here we describe briefly a holographic solar concentrator [124]. A compound holographic solar concentrator has been built and comprise several modules. Each module consists of two off axis hololenses and three PV cells in the configuration: Si-InGaP-Si.

Hololenses have been fabricated with DCG. The behavior or response of the hololens in relation to the wavelength is as follows. Light with a wavelength of 850 nm and higher wavelengths are transmitted without deviation through the hololens to a silicon PV cell that is behind the hololens. Light with a wavelength of about 500 nm or a little higher is sent to the InGaP cell that is off axis of the lens. With this method, the efficiency of the concentrator was 19% higher when compared to the more efficient cell system based only on Si cells.

Other optical configurations of holographic solar concentrators and related matter can be found in references [125,126,127].

### 4.7. Display Holography

The history of the intensive application of gelatin-containing photosensitive media in holography has been developed for more than fifty years, beginning with the pioneering work of Yu.N. Denisyuk [128] and E. Leith and Yu. Upatnieks [129]. They proposed methods for recording volume and transmissive holograms on silver halide photographic emulsions.

Gelatin-light sensitive materials in holography have been applied to fabricate displays used in industry, engineering, medicine, energy, education, advertising, metrology, microscopy, nondestructive testing, security holograms, displays of historical items and artistic subjects to mention but a few. Conferences dedicated to display holography have been developed since some decades ago. For example, the SPIE annual conference dedicated to holography is named “Practical Holography” this year (2018) is the XXXII session [130]. Other conference is named International Symposium on Display Holography which began 1982. This year the 11th conference will take place in Portugal [131]. Other conferences have been held in Russia like the Conferences dedicated to Prof. Yu. N. Denisyuk in St. Petersburg, for example [132]. Another conference that has been running since 2004 is named Russia Holo Expo, Holography, Science and Practice. This year it will be held in Nizhny Novgorod [133]. In the conferences, the subjects included: materials’ holographic performance and optical properties, modeling and analysis of holographic components, durability and environmental testing of materials and devices, improved processing materials, real-time holograms, applications of new materials in display holography and more.

Among the holographic displays are the ones that show museums’ works of art. These holograms were shown in travelling exhibitions. In the past heavy equipment like holographic tables, lasers and more were used to fabricate these holograms. Later, mobile apparatus for recording holograms were proposed. One of them [134] used a pulsed laser giving light with a wavelength of 526 nm, the energy per pulse was 1 J, pulse width 30 ns and coherence length of 1 m. FPR holographic plates were used (Slavich). With this camera, it is possible to record hologram portraits, objects of art, holograms for medical purposes, holographic logos and more.

Since 1995 a new camera and film [135] began to be developed to record holograms of museum’s work of art. The camera was ended in 2009. The new film was named Ultimate. Size of the hologram could be as large as 32 cm × 43 cm. It can give color holograms. To record the hologram three lasers giving light with the following wavelengths: 639 nm, 532 nm and 473 nm, were used. The camera weighted about 12 kg.

## 5. Conclusions

A review of works reflecting the properties of plain gelatin and light-sensitive medium based on gelatin, as well as examples of the use of these materials, allow us to conclude the following.

Plain gelatin is quite sensitive to the effects of short wave UV radiation, infrared and ionizing radiation. It is also sensitive to physical parameters like relative humidity.

Gelatin has a high compatibility with a wide variety of substances from simple mineral salts (silver halides, dichromates, etc.) and ending with complex organic compounds (dyes, for example). This allows gelatin to be sensitive to a wide range of electromagnetic radiation from UV to mid-infrared light, to present high resolution (up to 5000 L/mm), high power sensitivity and low noise.

Structure of gelatin can be changed under the influences (radiation or chemical treatment) to suffer structuring (tanning) or destruction.

These properties make plain gelatin and gelatin based light-sensitive materials indispensable for: its use as sensor, the recording of optical information, creating a variety of diffractive optical elements, including holographic optical elements and others.

## Figures and Tables

**Table 1:** Parameters of the regular holographic structures on silver halide photoemulsions and DCG.

#	Type of Gelatin-Containing Recording Medium	Layer Thickness, μm	Type of Structure	Maximum Achieved Value of the Height of the Relief *h*_max_, μm	Maximum Obtained Diffraction Efficiency η_1max_, % for λ = 0.6328 µm
**1**	Photoplates VRL Russia	14–18	Fresnel zone plate 0–57 L/mm	1.2–2	34
**2**	Photoplates VRL	14–18	Grating 110 L/mm	1.1	17
**3**	Photoplates VRL	14–18	Grating 110 L/mm	0.63	21.1
**4**	Photoplates PFG-01, Slavich	7	Grating 65 L/mm	1.35	28.5
**5**	Photoplates PFG-01	7	microlens array 10 L/mm	2.6–2.8	-
**6**	Photoplates Agfa-Gevaert 8E75	6–7	grating 40 L/mm	1.54	-
**7**	Photoplates Аgfa-Gevaert Millimask	5	Grating 130 L/mm	1.4	23
**8**	Photoplates Kodak HR	5	Grating 130 L/mm	1.2	25
**9**	Photoplates SRBSh (Kurchatov Institute of Atomic Energy), Russia	1.8	Grating 130 L/mm	-	24
**10**	DCG layer	51–86	Grating 103 L/mm	1.35–1.45	25
**11**	DCG layer	0.6–1.1	Grating 103 L/mm	0.6–0.9	28–30
**12**	Structure transfer from the DCG layer to the PMMA substrate	0.3–5	Grating 103 L/mm	0.48–1.3	8–25

**Table 2:** Parameters of holographic diffusers.

Sample No.	The Average Thickness of the Photographic Emulsions after Processing by the SWUV Method, µm	σ, µm	*h*_max_, µm	$\eta_{0},\;\%\;\mathrm{for}\;\lambda \;=\;0.6328\;µm$
**1**	1.4	0.54	1.9	0.12
**2**	2.4	0.36	1.5	0.079
**3**	3.4	0.41	1.8	0.057
